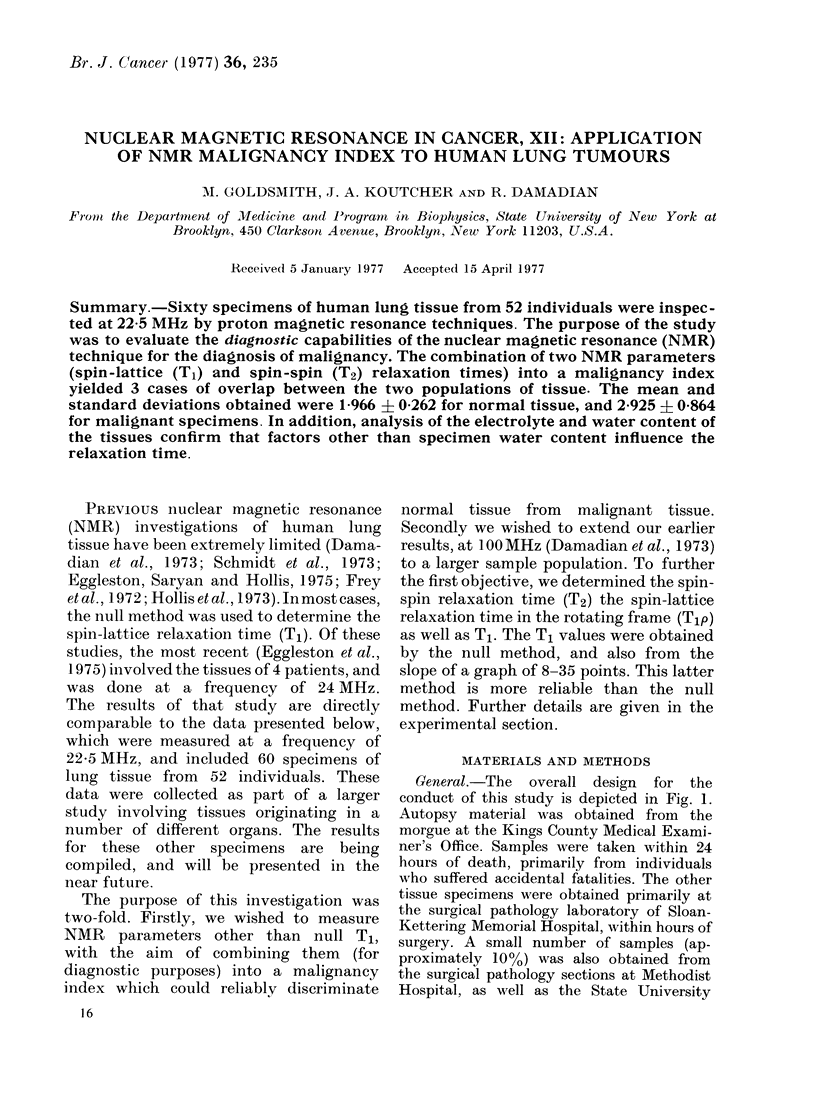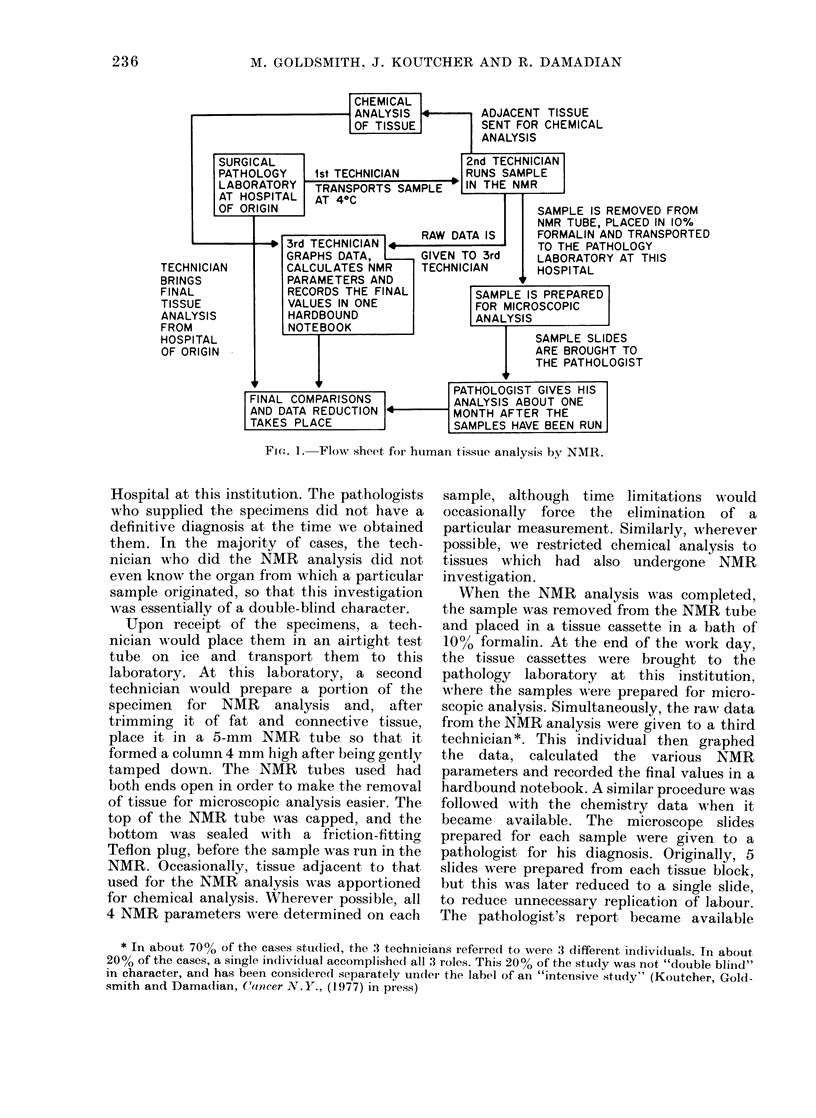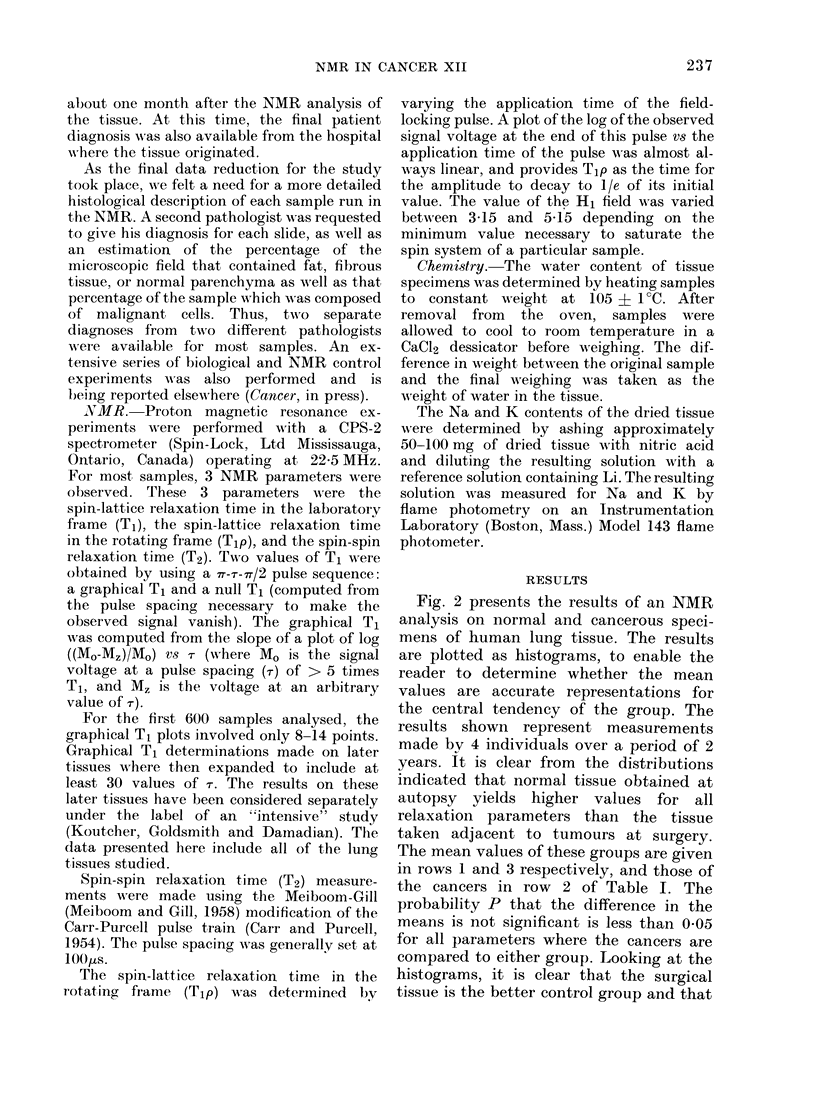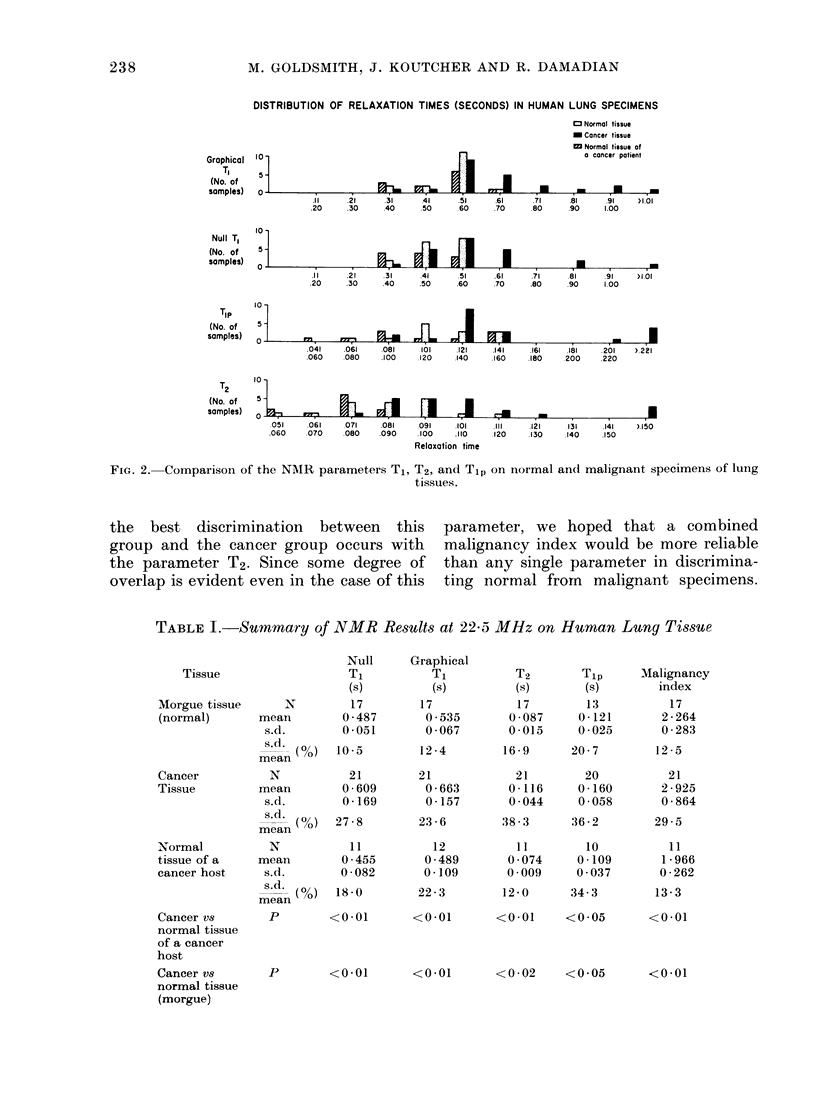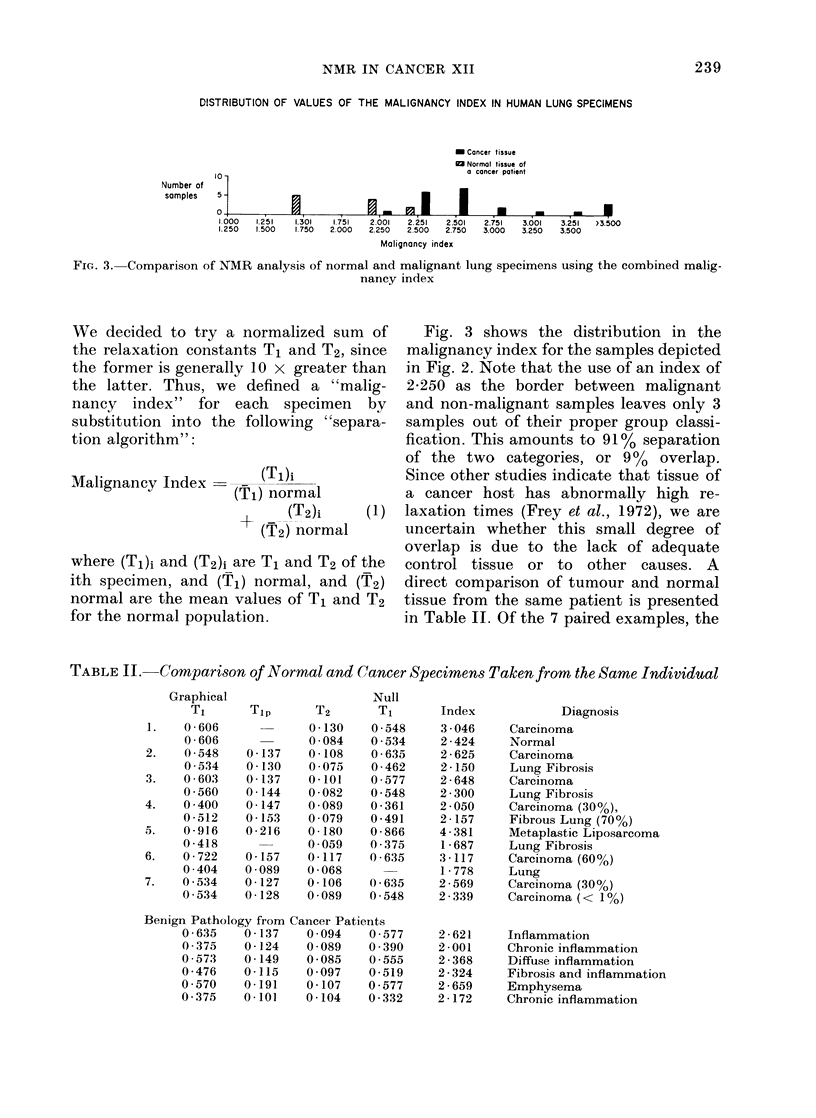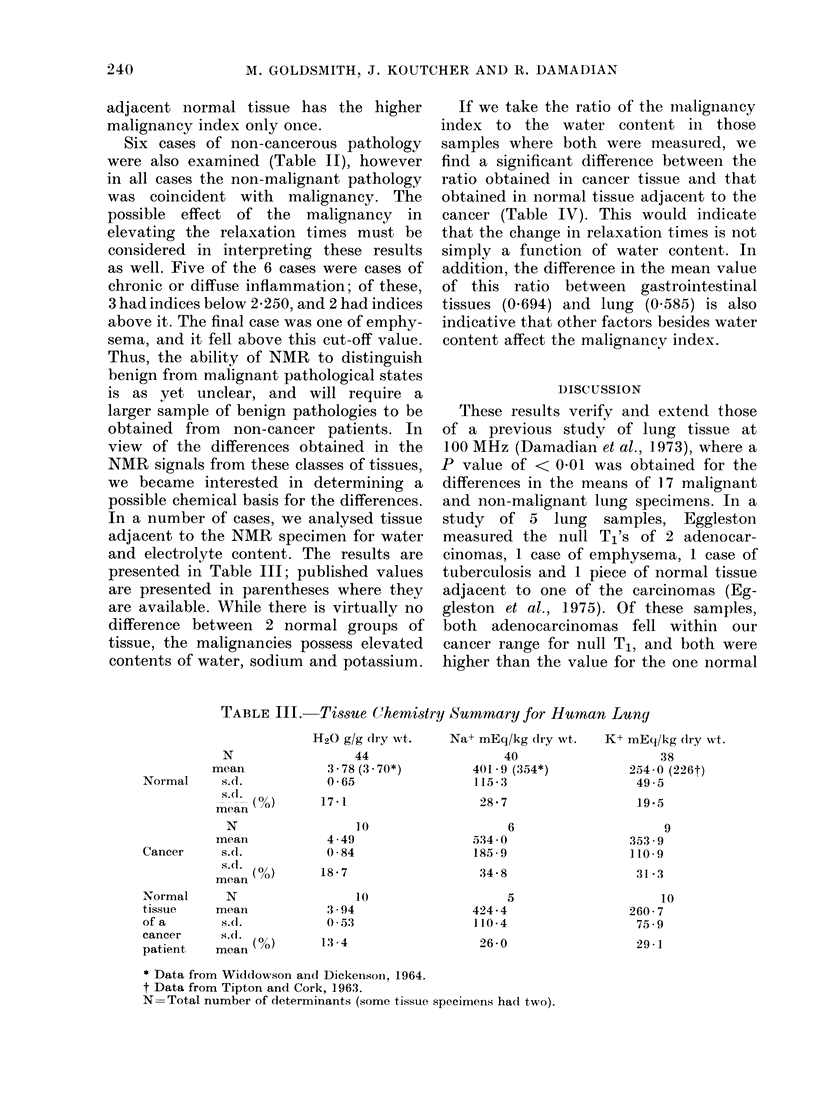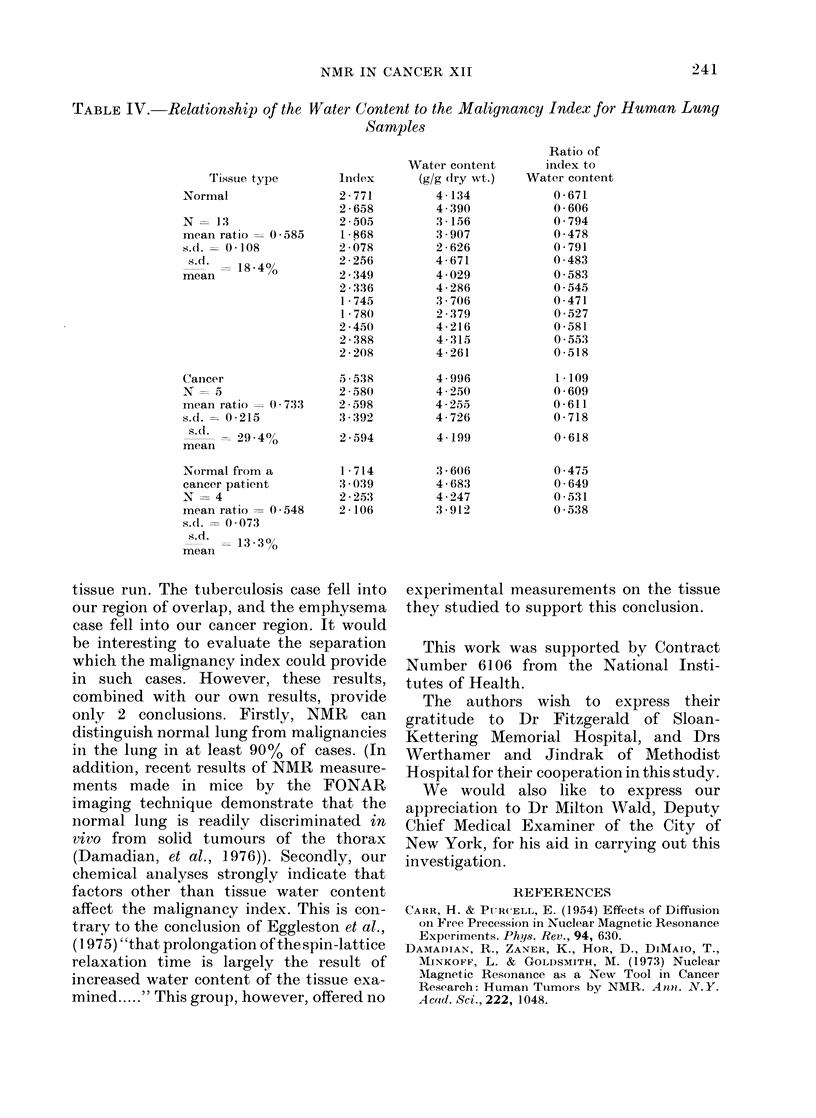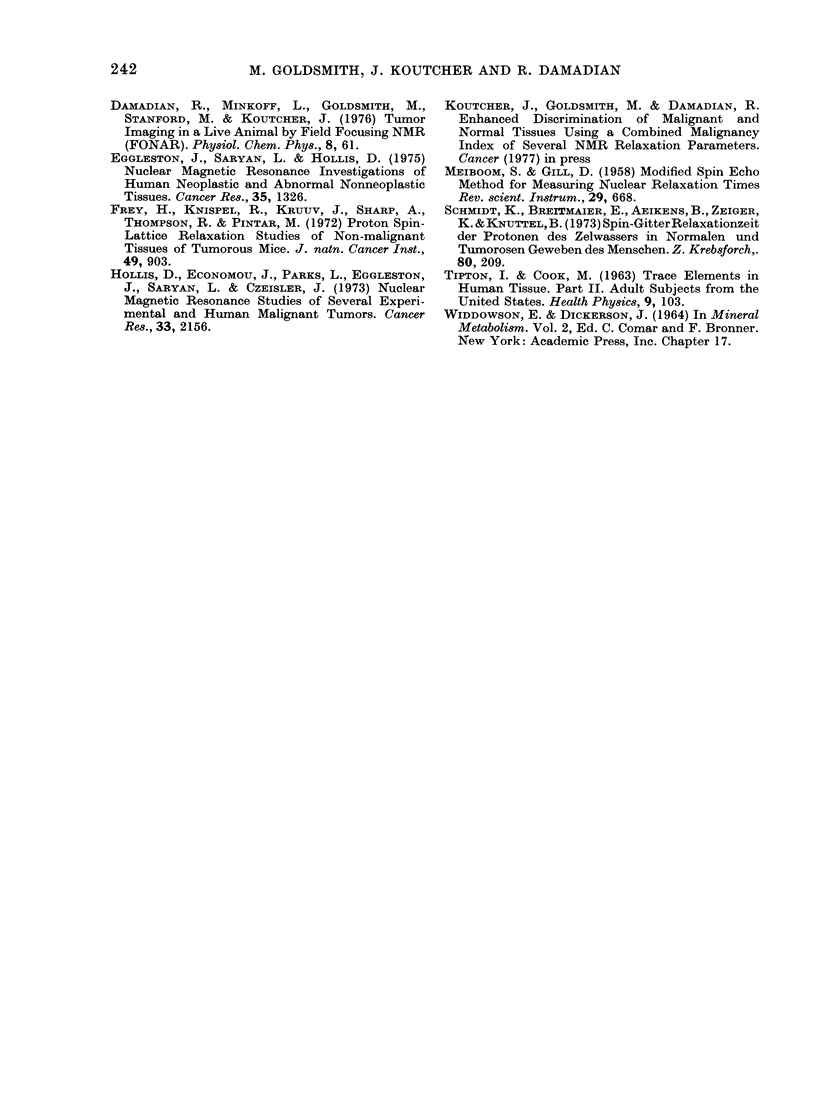# Nuclear magnetic resonance in cancer, XII: Application of NMR malignancy index to human lung tumours.

**DOI:** 10.1038/bjc.1977.183

**Published:** 1977-08

**Authors:** M. Goldsmith, J. A. Koutcher, R. Damadian

## Abstract

Sixty specimens of human lung tissue from 52 individuals were inspected at 22.5 MHz by proton magnetic resonance techniques. The purpose of the study was to evaluate the diagnostic capabilities of the nuclear magnetic resonance (NMR) technique for the diagnosis of malignancy. The combination of two NMR parameters (spin-lattice (T1) and spin-spin (T2) relaxation times) into a malignancy index yielded 3 cases of overlap between the two populations of tissue. The mean and standard deviations obtained were 1.966 +/- 0.262 for normal tissue, and 2.925 +/- 0.864 for malignant specimens. In addition, analysis of the electrolyte and water content of the tissues confirm that factors other than specimen water content influence the relaxation time.


*Br. J. Cancer* (1977) **36**, 235

### NUCLEAR MAGNETIC RESONANCE IN CANCER, XII: APPLICATION OF NMR MALIGNANCY INDEX TO HUMAN LUNG TUMOURS

M. GOLDSMITH, J. A. KOUTCHER AND R. DAMADIAN

*From the Department of Medicine and Program in Biophysics, State University of New York at Brooklyn, 450 Clarkson Avenue, Brooklyn, New York 11203, U.S.A.*

Received 5 January 1977 Accepted 15 April 1977

**Summary.—Sixty specimens of human lung tissue from 52 individuals were inspected at 22·5 MHz by proton magnetic resonance techniques. The purpose of the study was to evaluate the *diagnostic* capabilities of the nuclear magnetic resonance (NMR) technique for the diagnosis of malignancy. The combination of two NMR parameters (spin-lattice ($T_1$) and spin-spin ($T_2$) relaxation times) into a malignancy index yielded 3 cases of overlap between the two populations of tissue. The mean and standard deviations obtained were 1·966 $\pm$ 0·262 for normal tissue, and 2·925 $\pm$ 0·864 for malignant specimens. In addition, analysis of the electrolyte and water content of the tissues confirm that factors other than specimen water content influence the relaxation time.**

PREVIOUS nuclear magnetic resonance (NMR) investigations of human lung tissue have been extremely limited (Damadian *et al.*, 1973; Schmidt *et al.*, 1973; Eggleston, Saryan and Hollis, 1975; Frey *et al.*, 1972; Hollis *et al.*, 1973). In most cases, the null method was used to determine the spin-lattice relaxation time ($T_1$). Of these studies, the most recent (Eggleston *et al.*, 1975) involved the tissues of 4 patients, and was done at a frequency of 24 MHz. The results of that study are directly comparable to the data presented below, which were measured at a frequency of 22·5 MHz, and included 60 specimens of lung tissue from 52 individuals. These data were collected as part of a larger study involving tissues originating in a number of different organs. The results for these other specimens are being compiled, and will be presented in the near future.

The purpose of this investigation was two-fold. Firstly, we wished to measure NMR parameters other than null $T_1$, with the aim of combining them (for diagnostic purposes) into a malignancy index which could reliably discriminate normal tissue from malignant tissue. Secondly we wished to extend our earlier results, at 100 MHz (Damadian *et al.*, 1973) to a larger sample population. To further the first objective, we determined the spin-spin relaxation time ($T_2$) the spin-lattice relaxation time in the rotating frame ($T_{1\rho}$) as well as $T_1$. The $T_1$ values were obtained by the null method, and also from the slope of a graph of 8–35 points. This latter method is more reliable than the null method. Further details are given in the experimental section.

### MATERIALS AND METHODS

*General.*—The overall design for the conduct of this study is depicted in Fig. 1. Autopsy material was obtained from the morgue at the Kings County Medical Examiner's Office. Samples were taken within 24 hours of death, primarily from individuals who suffered accidental fatalities. The other tissue specimens were obtained primarily at the surgical pathology laboratory of Sloan-Kettering Memorial Hospital, within hours of surgery. A small number of samples (approximately 10%) was also obtained from the surgical pathology sections at Methodist Hospital, as well as the State University

FIG. 1.—Flow sheet for human tissue analysis by NMR.

Hospital at this institution. The pathologists who supplied the specimens did not have a definitive diagnosis at the time we obtained them. In the majority of cases, the technician who did the NMR analysis did not even know the organ from which a particular sample originated, so that this investigation was essentially of a double-blind character.

Upon receipt of the specimens, a technician would place them in an airtight test tube on ice and transport them to this laboratory. At this laboratory, a second technician would prepare a portion of the specimen for NMR analysis and, after trimming it of fat and connective tissue, place it in a 5-mm NMR tube so that it formed a column 4 mm high after being gently tamped down. The NMR tubes used had both ends open in order to make the removal of tissue for microscopic analysis easier. The top of the NMR tube was capped, and the bottom was sealed with a friction-fitting Teflon plug, before the sample was run in the NMR. Occasionally, tissue adjacent to that used for the NMR analysis was apportioned for chemical analysis. Wherever possible, all 4 NMR parameters were determined on each sample, although time limitations would occasionally force the elimination of a particular measurement. Similarly, wherever possible, we restricted chemical analysis to tissues which had also undergone NMR investigation.

When the NMR analysis was completed, the sample was removed from the NMR tube and placed in a tissue cassette in a bath of 10% formalin. At the end of the work day, the tissue cassettes were brought to the pathology laboratory at this institution, where the samples were prepared for microscopic analysis. Simultaneously, the raw data from the NMR analysis were given to a third technician*. This individual then graphed the data, calculated the various NMR parameters and recorded the final values in a hardbound notebook. A similar procedure was followed with the chemistry data when it became available. The microscope slides prepared for each sample were given to a pathologist for his diagnosis. Originally, 5 slides were prepared from each tissue block, but this was later reduced to a single slide, to reduce unnecessary replication of labour. The pathologist's report became available

* In about 70% of the cases studied, the 3 technicians referred to were 3 different individuals. In about 20% of the cases, a single individual accomplished all 3 roles. This 20% of the study was not "double blind" in character, and has been considered separately under the label of an "intensive study" (Koutcher, Goldsmith and Damadian, *Cancer N.Y.*, (1977) in press)

about one month after the NMR analysis of the tissue. At this time, the final patient diagnosis was also available from the hospital where the tissue originated.

As the final data reduction for the study took place, we felt a need for a more detailed histological description of each sample run in the NMR. A second pathologist was requested to give his diagnosis for each slide, as well as an estimation of the percentage of the microscopic field that contained fat, fibrous tissue, or normal parenchyma as well as that percentage of the sample which was composed of malignant cells. Thus, two separate diagnoses from two different pathologists were available for most samples. An extensive series of biological and NMR control experiments was also performed and is being reported elsewhere (*Cancer*, in press).

*NMR.*—Proton magnetic resonance experiments were performed with a CPS-2 spectrometer (Spin-Lock, Ltd Mississauga, Ontario, Canada) operating at 22·5 MHz. For most samples, 3 NMR parameters were observed. These 3 parameters were the spin-lattice relaxation time in the laboratory frame ($T_1$), the spin-lattice relaxation time in the rotating frame ($T_{1\rho}$), and the spin-spin relaxation time ($T_2$). Two values of $T_1$ were obtained by using a $\pi$-$\tau$-$\pi/2$ pulse sequence: a graphical $T_1$ and a null $T_1$ (computed from the pulse spacing necessary to make the observed signal vanish). The graphical $T_1$ was computed from the slope of a plot of log $((M_0\text{-}M_z)/M_0)$ *vs* $\tau$ (where $M_0$ is the signal voltage at a pulse spacing ($\tau$) of $> 5$ times $T_1$, and $M_z$ is the voltage at an arbitrary value of $\tau$).

For the first 600 samples analysed, the graphical $T_1$ plots involved only 8–14 points. Graphical $T_1$ determinations made on later tissues where then expanded to include at least 30 values of $\tau$. The results on these later tissues have been considered separately under the label of an "intensive" study (Koutcher, Goldsmith and Damadian). The data presented here include all of the lung tissues studied.

Spin-spin relaxation time ($T_2$) measurements were made using the Meiboom-Gill (Meiboom and Gill, 1958) modification of the Carr-Purcell pulse train (Carr and Purcell, 1954). The pulse spacing was generally set at $100\mu s$.

The spin-lattice relaxation time in the rotating frame ($T_{1\rho}$) was determined by varying the application time of the field-locking pulse. A plot of the log of the observed signal voltage at the end of this pulse *vs* the application time of the pulse was almost always linear, and provides $T_{1\rho}$ as the time for the amplitude to decay to $1/e$ of its initial value. The value of the $H_1$ field was varied between 3·15 and 5·15 depending on the minimum value necessary to saturate the spin system of a particular sample.

*Chemistry.*—The water content of tissue specimens was determined by heating samples to constant weight at $105 \pm 1°C$. After removal from the oven, samples were allowed to cool to room temperature in a $CaCl_2$ dessicator before weighing. The difference in weight between the original sample and the final weighing was taken as the weight of water in the tissue.

The Na and K contents of the dried tissue were determined by ashing approximately 50–100 mg of dried tissue with nitric acid and diluting the resulting solution with a reference solution containing Li. The resulting solution was measured for Na and K by flame photometry on an Instrumentation Laboratory (Boston, Mass.) Model 143 flame photometer.

### RESULTS

Fig. 2 presents the results of an NMR analysis on normal and cancerous specimens of human lung tissue. The results are plotted as histograms, to enable the reader to determine whether the mean values are accurate representations for the central tendency of the group. The results shown represent measurements made by 4 individuals over a period of 2 years. It is clear from the distributions indicated that normal tissue obtained at autopsy yields higher values for all relaxation parameters than the tissue taken adjacent to tumours at surgery. The mean values of these groups are given in rows 1 and 3 respectively, and those of the cancers in row 2 of Table I. The probability *P* that the difference in the means is not significant is less than 0·05 for all parameters where the cancers are compared to either group. Looking at the histograms, it is clear that the surgical tissue is the better control group and that

FIG. 2.—Comparison of the NMR parameters $T_1$, $T_2$, and $T_{1p}$ on normal and malignant specimens of lung tissues.

the best discrimination between this group and the cancer group occurs with the parameter $T_2$. Since some degree of overlap is evident even in the case of this parameter, we hoped that a combined malignancy index would be more reliable than any single parameter in discriminating normal from malignant specimens.

TABLE I.—*Summary of NMR Results at 22·5 MHz on Human Lung Tissue*

| Tissue | | Null $T_1$ (s) | Graphical $T_1$ (s) | $T_2$ (s) | $T_{1p}$ (s) | Malignancy index |
|---|---|---|---|---|---|---|
| Morgue tissue (normal) | N | 17 | 17 | 17 | 13 | 17 |
| | mean | 0·487 | 0·535 | 0·087 | 0·121 | 2·264 |
| | s.d. | 0·051 | 0·067 | 0·015 | 0·025 | 0·283 |
| | $\frac{\text{s.d.}}{\text{mean}}$ (%) | 10·5 | 12·4 | 16·9 | 20·7 | 12·5 |
| Cancer Tissue | N | 21 | 21 | 21 | 20 | 21 |
| | mean | 0·609 | 0·663 | 0·116 | 0·160 | 2·925 |
| | s.d. | 0·169 | 0·157 | 0·044 | 0·058 | 0·864 |
| | $\frac{\text{s.d.}}{\text{mean}}$ (%) | 27·8 | 23·6 | 38·3 | 36·2 | 29·5 |
| Normal tissue of a cancer host | N | 11 | 12 | 11 | 10 | 11 |
| | mean | 0·455 | 0·489 | 0·074 | 0·109 | 1·966 |
| | s.d. | 0·082 | 0·109 | 0·009 | 0·037 | 0·262 |
| | $\frac{\text{s.d.}}{\text{mean}}$ (%) | 18·0 | 22·3 | 12·0 | 34·3 | 13·3 |
| Cancer *vs* normal tissue of a cancer host | *P* | $<0·01$ | $<0·01$ | $<0·01$ | $<0·05$ | $<0·01$ |
| Cancer *vs* normal tissue (morgue) | *P* | $<0·01$ | $<0·01$ | $<0·02$ | $<0·05$ | $<0·01$ |

FIG. 3.—Comparison of NMR analysis of normal and malignant lung specimens using the combined malignancy index

We decided to try a normalized sum of the relaxation constants $T_1$ and $T_2$, since the former is generally 10 × greater than the latter. Thus, we defined a "malignancy index" for each specimen by substitution into the following "separation algorithm":

$$\text{Malignancy Index} = \frac{(T_1)_i}{(\bar{T}_1)\ \text{normal}} + \frac{(T_2)_i}{(\bar{T}_2)\ \text{normal}} \qquad (1)$$

where $(T_1)_i$ and $(T_2)_i$ are $T_1$ and $T_2$ of the ith specimen, and $(\bar{T}_1)$ normal, and $(\bar{T}_2)$ normal are the mean values of $T_1$ and $T_2$ for the normal population.

Fig. 3 shows the distribution in the malignancy index for the samples depicted in Fig. 2. Note that the use of an index of 2·250 as the border between malignant and non-malignant samples leaves only 3 samples out of their proper group classification. This amounts to 91% separation of the two categories, or 9% overlap. Since other studies indicate that tissue of a cancer host has abnormally high relaxation times (Frey *et al.*, 1972), we are uncertain whether this small degree of overlap is due to the lack of adequate control tissue or to other causes. A direct comparison of tumour and normal tissue from the same patient is presented in Table II. Of the 7 paired examples, the

TABLE II.—*Comparison of Normal and Cancer Specimens Taken from the Same Individual*

| | Graphical $T_1$ | $T_{1p}$ | $T_2$ | Null $T_1$ | Index | Diagnosis |
|---|---|---|---|---|---|---|
| 1. | 0·606 | — | 0·130 | 0·548 | 3·046 | Carcinoma |
| | 0·606 | — | 0·084 | 0·534 | 2·424 | Normal |
| 2. | 0·548 | 0·137 | 0·108 | 0·635 | 2·625 | Carcinoma |
| | 0·534 | 0·130 | 0·075 | 0·462 | 2·150 | Lung Fibrosis |
| 3. | 0·603 | 0·137 | 0·101 | 0·577 | 2·648 | Carcinoma |
| | 0·560 | 0·144 | 0·082 | 0·548 | 2·300 | Lung Fibrosis |
| 4. | 0·400 | 0·147 | 0·089 | 0·361 | 2·050 | Carcinoma (30%), |
| | 0·512 | 0·153 | 0·079 | 0·491 | 2·157 | Fibrous Lung (70%) |
| 5. | 0·916 | 0·216 | 0·180 | 0·866 | 4·381 | Metaplastic Liposarcoma |
| | 0·418 | — | 0·059 | 0·375 | 1·687 | Lung Fibrosis |
| 6. | 0·722 | 0·157 | 0·117 | 0·635 | 3·117 | Carcinoma (60%) |
| | 0·404 | 0·089 | 0·068 | — | 1·778 | Lung |
| 7. | 0·534 | 0·127 | 0·106 | 0·635 | 2·569 | Carcinoma (30%) |
| | 0·534 | 0·128 | 0·089 | 0·548 | 2·339 | Carcinoma (< 1%) |
| Benign Pathology from Cancer Patients | | | | | | |
| | 0·635 | 0·137 | 0·094 | 0·577 | 2·621 | Inflammation |
| | 0·375 | 0·124 | 0·089 | 0·390 | 2·001 | Chronic inflammation |
| | 0·573 | 0·149 | 0·085 | 0·555 | 2·368 | Diffuse inflammation |
| | 0·476 | 0·115 | 0·097 | 0·519 | 2·324 | Fibrosis and inflammation |
| | 0·570 | 0·191 | 0·107 | 0·577 | 2·659 | Emphysema |
| | 0·375 | 0·101 | 0·104 | 0·332 | 2·172 | Chronic inflammation |

adjacent normal tissue has the higher malignancy index only once.

Six cases of non-cancerous pathology were also examined (Table II), however in all cases the non-malignant pathology was coincident with malignancy. The possible effect of the malignancy in elevating the relaxation times must be considered in interpreting these results as well. Five of the 6 cases were cases of chronic or diffuse inflammation; of these, 3 had indices below 2·250, and 2 had indices above it. The final case was one of emphysema, and it fell above this cut-off value. Thus, the ability of NMR to distinguish benign from malignant pathological states is as yet unclear, and will require a larger sample of benign pathologies to be obtained from non-cancer patients. In view of the differences obtained in the NMR signals from these classes of tissues, we became interested in determining a possible chemical basis for the differences. In a number of cases, we analysed tissue adjacent to the NMR specimen for water and electrolyte content. The results are presented in Table III; published values are presented in parentheses where they are available. While there is virtually no difference between 2 normal groups of tissue, the malignancies possess elevated contents of water, sodium and potassium.

If we take the ratio of the malignancy index to the water content in those samples where both were measured, we find a significant difference between the ratio obtained in cancer tissue and that obtained in normal tissue adjacent to the cancer (Table IV). This would indicate that the change in relaxation times is not simply a function of water content. In addition, the difference in the mean value of this ratio between gastrointestinal tissues (0·694) and lung (0·585) is also indicative that other factors besides water content affect the malignancy index.

### DISCUSSION

These results verify and extend those of a previous study of lung tissue at 100 MHz (Damadian *et al.*, 1973), where a $P$ value of $< 0{\cdot}01$ was obtained for the differences in the means of 17 malignant and non-malignant lung specimens. In a study of 5 lung samples, Eggleston measured the null $T_1$'s of 2 adenocarcinomas, 1 case of emphysema, 1 case of tuberculosis and 1 piece of normal tissue adjacent to one of the carcinomas (Eggleston *et al.*, 1975). Of these samples, both adenocarcinomas fell within our cancer range for null $T_1$, and both were higher than the value for the one normal

TABLE III.—*Tissue Chemistry Summary for Human Lung*

| | | $H_2O$ g/g dry wt. | $Na^+$ mEq/kg dry wt. | $K^+$ mEq/kg dry wt. |
|---|---|---|---|---|
| Normal | N | 44 | 40 | 38 |
| | mean | 3·78 (3·70*) | 401·9 (354*) | 254·0 (226†) |
| | s.d. | 0·65 | 115·3 | 49·5 |
| | $\frac{\text{s.d.}}{\text{mean}}$ (%) | 17·1 | 28·7 | 19·5 |
| Cancer | N | 10 | 6 | 9 |
| | mean | 4·49 | 534·0 | 353·9 |
| | s.d. | 0·84 | 185·9 | 110·9 |
| | $\frac{\text{s.d.}}{\text{mean}}$ (%) | 18·7 | 34·8 | 31·3 |
| Normal tissue of a cancer patient | N | 10 | 5 | 10 |
| | mean | 3·94 | 424·4 | 260·7 |
| | s.d. | 0·53 | 110·4 | 75·9 |
| | $\frac{\text{s.d.}}{\text{mean}}$ (%) | 13·4 | 26·0 | 29·1 |

* Data from Widdowson and Dickenson, 1964.
† Data from Tipton and Cork, 1963.
N = Total number of determinants (some tissue specimens had two).

TABLE IV.—*Relationship of the Water Content to the Malignancy Index for Human Lung Samples*

| Tissue type | Index | Water content (g/g dry wt.) | Ratio of index to Water content |
|---|---|---|---|
| Normal | 2·771 | 4·134 | 0·671 |
| | 2·658 | 4·390 | 0·606 |
| N = 13 | 2·505 | 3·156 | 0·794 |
| mean ratio = 0·585 | 1·868 | 3·907 | 0·478 |
| s.d. = 0·108 | 2·078 | 2·626 | 0·791 |
| $\frac{\text{s.d.}}{\text{mean}} = 18 \cdot 4\%$ | 2·256 | 4·671 | 0·483 |
| | 2·349 | 4·029 | 0·583 |
| | 2·336 | 4·286 | 0·545 |
| | 1·745 | 3·706 | 0·471 |
| | 1·780 | 2·379 | 0·527 |
| | 2·450 | 4·216 | 0·581 |
| | 2·388 | 4·315 | 0·553 |
| | 2·208 | 4·261 | 0·518 |
| Cancer | 5·538 | 4·996 | 1·109 |
| N = 5 | 2·580 | 4·250 | 0·609 |
| mean ratio = 0·733 | 2·598 | 4·255 | 0·611 |
| s.d. = 0·215 | 3·392 | 4·726 | 0·718 |
| $\frac{\text{s.d.}}{\text{mean}} = 29 \cdot 4\%$ | 2·594 | 4·199 | 0·618 |
| Normal from a | 1·714 | 3·606 | 0·475 |
| cancer patient | 3·039 | 4·683 | 0·649 |
| N = 4 | 2·253 | 4·247 | 0·531 |
| mean ratio = 0·548 | 2·106 | 3·912 | 0·538 |
| s.d. = 0·073 | | | |
| $\frac{\text{s.d.}}{\text{mean}} = 13 \cdot 3\%$ | | | |

tissue run. The tuberculosis case fell into our region of overlap, and the emphysema case fell into our cancer region. It would be interesting to evaluate the separation which the malignancy index could provide in such cases. However, these results, combined with our own results, provide only 2 conclusions. Firstly, NMR can distinguish normal lung from malignancies in the lung in at least 90% of cases. (In addition, recent results of NMR measurements made in mice by the FONAR imaging technique demonstrate that the normal lung is readily discriminated *in vivo* from solid tumours of the thorax (Damadian, *et al.*, 1976)). Secondly, our chemical analyses strongly indicate that factors other than tissue water content affect the malignancy index. This is contrary to the conclusion of Eggleston *et al.*, (1975) "that prolongation of the spin-lattice relaxation time is largely the result of increased water content of the tissue examined....." This group, however, offered no experimental measurements on the tissue they studied to support this conclusion.

This work was supported by Contract Number 6106 from the National Institutes of Health.

The authors wish to express their gratitude to Dr Fitzgerald of Sloan-Kettering Memorial Hospital, and Drs Werthamer and Jindrak of Methodist Hospital for their cooperation in this study.

We would also like to express our appreciation to Dr Milton Wald, Deputy Chief Medical Examiner of the City of New York, for his aid in carrying out this investigation.

### REFERENCES

CARR, H. & PURCELL, E. (1954) Effects of Diffusion on Free Precession in Nuclear Magnetic Resonance Experiments. *Phys. Rev.*, **94**, 630.

DAMADIAN, R., ZANER, K., HOR, D., DIMAIO, T., MINKOFF, L. & GOLDSMITH, M. (1973) Nuclear Magnetic Resonance as a New Tool in Cancer Research: Human Tumors by NMR. *Ann. N.Y. Acad. Sci.*, **222**, 1048.

DAMADIAN, R., MINKOFF, L., GOLDSMITH, M., STANFORD, M. & KOUTCHER, J. (1976) Tumor Imaging in a Live Animal by Field Focusing NMR (FONAR). *Physiol. Chem. Phys.*, **8**, 61.

EGGLESTON, J., SARYAN, L. & HOLLIS, D. (1975) Nuclear Magnetic Resonance Investigations of Human Neoplastic and Abnormal Nonneoplastic Tissues. *Cancer Res.*, **35**, 1326.

FREY, H., KNISPEL, R., KRUUV, J., SHARP, A., THOMPSON, R. & PINTAR, M. (1972) Proton Spin-Lattice Relaxation Studies of Non-malignant Tissues of Tumorous Mice. *J. natn. Cancer Inst.*, **49**, 903.

HOLLIS, D., ECONOMOU, J., PARKS, L., EGGLESTON, J., SARYAN, L. & CZEISLER, J. (1973) Nuclear Magnetic Resonance Studies of Several Experimental and Human Malignant Tumors. *Cancer Res.*, **33**, 2156.

KOUTCHER, J., GOLDSMITH, M. & DAMADIAN, R. Enhanced Discrimination of Malignant and Normal Tissues Using a Combined Malignancy Index of Several NMR Relaxation Parameters. *Cancer* (1977) in press

MEIBOOM, S. & GILL, D. (1958) Modified Spin Echo Method for Measuring Nuclear Relaxation Times *Rev. scient. Instrum.*, **29**, 668.

SCHMIDT, K., BREITMAIER, E., AEIKENS, B., ZEIGER, K. & KNUTTEL, B. (1973) Spin-Gitter Relaxationzeit der Protonen des Zelwassers in Normalen und Tumorosen Geweben des Menschen. *Z. Krebsforch*,. **80**, 209.

TIPTON, I. & COOK, M. (1963) Trace Elements in Human Tissue. Part II. Adult Subjects from the United States. *Health Physics*, **9**, 103.

WIDDOWSON, E. & DICKERSON, J. (1964) In *Mineral Metabolism*. Vol. 2, Ed. C. Comar and F. Bronner. New York: Academic Press, Inc. Chapter 17.